# Acute effects of high‐intensity interval training session and endurance exercise on pulmonary function and cardiorespiratory coupling

**DOI:** 10.14814/phy2.14455

**Published:** 2020-08-03

**Authors:** David C. Andrade, Alexis Arce‐Alvarez, Felipe Parada, Sebastian Uribe, Pamela Gordillo, Anita Dupre, Carla Ojeda, Fiorella Palumbo, Guillermo Castro, Manuel Vasquez‐Muñoz, Rodrigo Del Rio, Rodrigo Ramirez‐Campillo, Mikel Izquierdo

**Affiliations:** ^1^ Centro de Investigación en Fisiología del Ejercicio Facultad de Ciencias Universidad Mayor Santiago Chile; ^2^ Pedagogía en Educación Física, Deportes y Recreación Universidad Mayor Santiago Chile; ^3^ Laboratory of Cardiorespiratory Control Department of Physiology Pontificia Universidad Católica de Chile Santiago Chile; ^4^ Escuela de Kinesiología Facultad de Salud Universidad Católica Silva Henríquez Santiago Chile; ^5^ Navarrabiomed Complejo Hospitalario de Navarra (CHN)‐Universidad Pública de Navarra (UPNA) IDISNA Pamplona Spain; ^6^ Unidad de Estadística Departamento de Calidad Clínica Santa María Santiago Chile; ^7^ Centro de Envejecimiento y Regeneración (CARE) Pontificia Universidad Católica de Chile Santiago Chile; ^8^ Centro de Excelencia en Biomedicina de Magallanes (CEBIMA) Universidad de Magallanes Punta Arenas Chile; ^9^ Laboratory of Human Performance. Quality of Life and Wellness Research Group Department of Physical Activity Sciences Universidad de Los Lagos Osorno Chile; ^10^ CIBER of Frailty and Healthy Aging (CIBERFES) Instituto de Salud Carlos III Madrid Spain

**Keywords:** autonomic control, cardiorespiratory coupling, endurance exercise, exercise training, high‐ intensity interval training

## Abstract

The aim of this study was to determine the acute effects of high‐intensity interval training (HIIT) exercise and endurance exercise (EE) on pulmonary function, sympathetic/parasympathetic balance, and cardiorespiratory coupling (CRC) in healthy participants. Using a crossover repeated‐measurements design, four females and four males were exposed to EE (20 min at 80% maximal heart rate [HR]), HIIT (1 min of exercise at 90% maximal HR per 1 min of rest, 10 times), or control condition (resting). Pulmonary function, HR, CRC, and heart rate variability (HRV) were assessed before and after the interventions. Results revealed no significant effects of EE or HIIT on pulmonary function. The EE, but not HIIT, significantly increased CRC. In contrast, HRV was markedly changed by HIIT, not by EE. Indeed, both the low‐frequency (LF_HRV_) and high‐frequency (HF_HRV_) components of HRV were increased and decreased, respectively, after HIIT. The increase in LF_HRV_ was greater after HIIT than after EE. Therefore, a single bout of HIIT or EE has no effects on pulmonary function. Moreover, CRC and cardiac autonomic regulation are targeted differently by the two exercise modalities.

## 1 INTRODUCTION

It is known that both endurance exercise (EE) and high‐intensity interval training (HIIT) have a positive impact on central and peripheral adaptations associated with aerobic and anaerobic fitness (Gibala et al., 2006; MacInnis & Gibala, 2017). Also, EE may improve body composition, metabolic risk factors, cardiac autonomic control, and maximal aerobic capacity (Hottenrott, Ludyga, & Schulze, 2012; Rivera‐Brown & Frontera, 2012). Several mechanisms associated with these beneficial effects are related to favorable changes in anti‐inflammatory and intramuscular glycogen metabolism, increased capillary and mitochondrial density in skeletal muscle, and increased oxidation of free fatty acids (Alansare, Alford, Lee, Church, & Jung, 2018; Ismail, Keating, Baker, & Johnson, 2012). High‐intensity interval training is widely used as an effective method to improve maximal aerobic capacity, sympathovagal balance, and oxidative capacity in the skeletal muscle (Alansare et al., 2018; Hoshino, Kitaoka Y, & Hatta, 2016). However, while both HIIT and EE elicit comparable physiological adaptations, the acute responses to these exercise protocols has not been completely described and in fact, could be different. Indeed, compared with EE, the total amount of energy expenditure in a typical HIIT session is significantly lower (Skelly et al., 2014). Moreover, there is limited evidence on the effects of a single exercise session of HIIT and EE on pulmonary function, autonomic regulation, and cardiorespiratory coupling (CRC).

Heart activity and breathing pattern can be viewed as a nonlinear dynamical system based on two coupled oscillators: heart rate and breathing time series (Scholkmann & Wolf, 2019). This phenomenon is usually referred to as CRC and involves a complex interplay between the activity of the brainstem (sympathetic and parasympathetic nucleus and central respiratory group) and the heart and lungs series (Scholkmann & Wolf, 2019), which could contribute to energy efficiency of the cardiorespiratory system. It has been shown that CRC increases in healthy individuals during an acute bout of moderate‐intensity (i.e., endurance‐type) exercise as determined through cross‐correlation analysis (Santos, Lopes, Jandre, & Giannella‐Neto, 2010). However, evidence is lacking about the effects of a single bout of HIIT or EE on CRC. Considering that coupled oscillations (heart–lung) are affected by the autonomic nervous system, it is possible that exercise at different intensities (i.e., endurance and HIIT) could modify the sympatho‐vagal balance at the brainstem level, affecting heart rate and breathing function, which consequently impact CRC. Therefore, the aim of this study was to compare the acute effects of HIIT and EE on pulmonary function, autonomic balance determined through heart rate variability (HRV), and CRC estimated through mutual information theory and phase models, in healthy participants.

## 2 METHODS

### 2.1 Subjects

Eight physically active participants (recreational runners; four male and four female) were recruited in the present study: height: 1.7 ± 0.1 m; body mass: 63.3 ± 5.7 kg; body mass index (BMI): 22.0 ± 2.4 kg/m^2^; age: 23.9 ± 3.1 years). All female were measured in the same stage of menstrual cycle in different weeks. Exercise sessions were conducted between 13:00 and 17:00 hr and subjects refrained from drinking alcohol, smoke, caffeine, or drugs that alter autonomic control 48 hr before exercise session. To assess the effects of a single bout of HIIT and EE, as independent variables, on pulmonary function, cardiovascular responses, autonomic control, and cardiorespiratory coupling, the same subjects were randomly assigned to HIIT, EE, and control conditions. Accordingly, this was a mixed crossover, repeated‐measures design. Participants were carefully informed about the experiment procedures, and the possible risks and benefits associated with their participation in the study. An appropriate signed informed consent document was obtained in accordance with the latest version of the Declaration of Helsinki. Separated by 7 days for men and accordingly to menstrual cycle of the female, participants completed an acute bout of HIIT (Francois & Little, 2015), EE (Corte de Araujo et al., 2012), or a control condition (20 min of resting standing on treadmill), on a treadmill (Power Jog, J100800 Cardio Sport running machine, North Charleston, SC).

### 2.2 Experimental procedure

**FIGURE 1:**
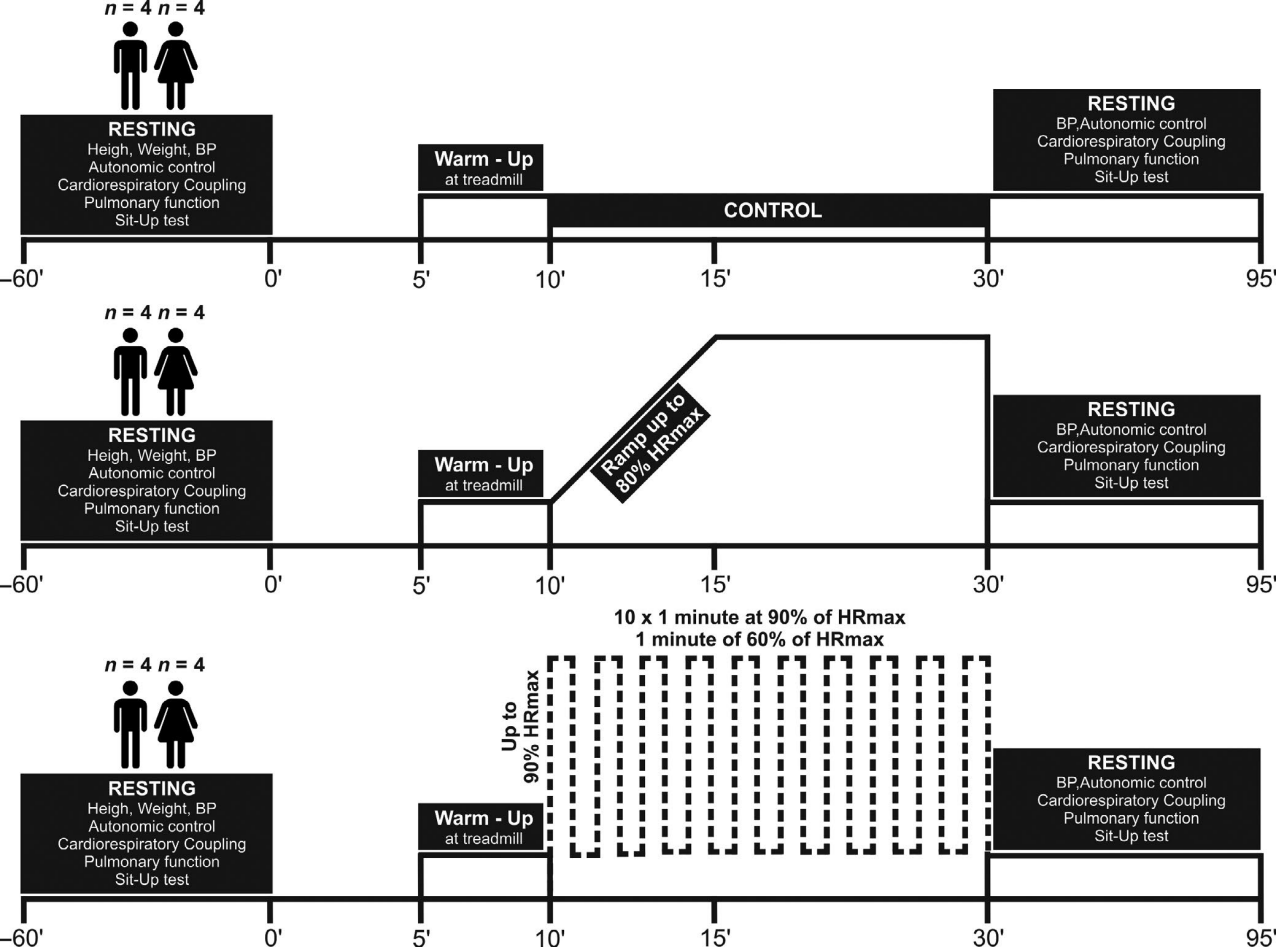
Experimental design. Physically active men (*n* = 4) and women (*n* = 4), performed 3 different randomly ordered protocols (HIIT, endurance exercise [EE] and control) in a treadmill. Before and after each experimental procedure individuals were assessed on: blood pressure, autonomic control, cardiorespiratory coupling, pulmonary function and orthostatic sit‐up test. HRmax, maximal heart rate

A schematic representation of the experimental study design is depicted in Figure 1.

None of the participants had any background (in the 6‐month period preceding the study) in regular strength training or competitive sports activity. Exclusion criteria considered for enrollment were: (a) potential medical problems or a history of ankle, knee, or back injury; (b) any lower extremity reconstructive surgery in the past 2 years or unresolved musculoskeletal disorders; (c) autonomic control impairment at rest, estimated by HRV disturbances (low to high frequency ratio of HRV <2.3) (Nunan, Sandercock, & Brodie, 2010); and (d) history of chronic obstructive or restrictive pulmonary diseases and/or altered spirometry on the day of the preexercise session (forced expiratory volume at first second (FEV1)/ vital capacity (VC) <70, FEV1 <80% of predicted value or VC <80% of predicted value).

Participants were carefully familiarized with the tests procedures before the measurements were taken. Randomly the type of exercise of session was chosen (HIIT, EE, or control). All participants were subject at the same warm‐up muscle actions prior to the exercises (Andrade et al., 2015). The warm‐up consisted of running at 6–7 km/hr on a treadmill (Power Jog). Tests were always administered in the same order (clinical spirometry, electrocardiogram, blood pressure, and sit‐up test, Figure 1), time of day (between 13:00 and 17:00 hr), and by the same investigators. The day before each experimental condition, participants were instructed to (a) have a good night sleep (~8 hr) and (b) use the same athletic shoes and clothing during the protocols. HIIT consisted in 1 min of exercise at 90% of maximal heart rate (HRmax) and 1 min of rest, repeated 10 times (Francois & Little, 2015); EE involved 5 min to ramp‐up to 80% of HRmax followed by 20 min at 80% HRmax (Corte de Araujo et al., 2012); and control involved 20 min of resting. HR during exercise was monitored with a telemetry device (Polar, V800, Finland).

Prior to, and after each experimental condition, height, body mass, systolic (SBP) and diastolic (DBP) blood pressure, mean arterial blood pressure (MABP), pulse pressure (PP), HR, clinical spirometry (VC; peak expiratory flow [PEF]; peak inspiratory flow [PIF]; FEV1; FEV1/VC; forced expiratory flow at 25% [FEF 25], 50% [FEF 50] and 75% [FEF 75] of VC), and the orthostatic sit‐up test measurements were taken. Height was measured using a wall‐mounted stadiometer (HR‐200, Tanita, Japan) recorded to the nearest 0.1 cm. Body mass was measured to the nearest 0.1 kg using a digital scale (BF‐350, Tanita, IL, USA). BMI was calculated as body mass/height^2^.

### 2.3 Pulmonary function

Pulmonary function was assessed according to the ATS/ERS Task Force consensus (Miller et al., 2005). Briefly, from the tidal volume, subjects were asked to perform a maximal inspiration (inspiratory reserve volume), return to tidal volume, and then perform a maximal expiration (expiratory reserve volume). We used the maximal expiratory curve to calculate VC, PEF, PIF, FEV1, FEV1/VC, FEF 25, FEF 50, and FEF 75. All recordings were performed with a clinical spirometry, calibrated according to the manufacturer instructions (Jaeger, Vyaire Medical Care).

### 2.4 Continuous recording of ventilation and electrocardiogram

In addition to pulmonary function assessments, prior to and after each experimental condition, ventilatory flow and a 2‐lead ECG (10 min at rest pre and post exercise and during sit‐up test) were recorded following the orthostatic sit‐up test (20 min total time), similar to a previous study (Currie, Wong, Warburton, & Krassioukov, 2015). Briefly, subjects were asked to maintain a supine position (10 min) and then asked to change to an upright position as slowly and as smoothly as possible (10 min) (Currie et al., 2015). The heart rate (HR) response and R‐R time series (5 min before orthostatic sit‐up test and 5 min after orthostatic sit‐up test) were used to estimate sympathoexcitation (see Autonomic control methods section). All recordings were sampled at 1 kHz with an analogic‐digital recording system (ADInstruments). The HR and ventilatory flow were analyzed with LabChart Pro 8.0 (ADInstruments).

### 2.5 Arterial blood pressure and oxygen saturation

Prior to, and after each experimental condition, SBP and DBP were determined. From SBP and DBP, MABP (1/3 of SBP + 2/3 of DBP) and PP (SBP‐DBP) were calculated as previously described (Álvarez et al., 2013). Measurements were determined with a sphygmomanometer (Tenso Medical Instruments Co., Ltd.) and a stethoscope (Littmann Cardiology, 3M, Bracknell, UK) by the same experienced clinician. In addition, SpO_2_ was determined before and after each acute exercise session (BuleTooth PULSE OXIMETER, BK‐P02, China).

### 2.6 Heart rate variability

Heart rate variability was used as an indirect measure of autonomic balance of the heart (Camm, Malik, & Bigger, 1996). From the ECG recordings, time series were obtained and autoregressive algorithm with Hann windowing of 50% overlap was used to obtain the power spectral density (PSD) of HRV. Cut‐off frequencies were defined as: very low frequency: 0.00–0.04; low frequency (LF_HRV_): 0.04–0.15 Hz and high frequency (HF_HRV_) 0.15–0.45 Hz (Yuda et al., 2018). Additionally, we used the LF/HF_HRV_ ratio as an indicator of autonomic balance of the heart. LF_HRV_ and HF_HRV_ were expressed as normalized units (n.u.) and raw dada. Analysis was performed within in a 10 min window using Kubios HRV Premium Software v3.1 (Kubios, Kuopio Finland).

In addition, to estimate the overall autonomic disruption after the interventions, spectral non‐stationary analysis was used (2 s resolution) during the orthostatic sit‐up test. The LF_HRV_ component was used as indicator of autonomic deregulation. Quantification was performed from the area under the curve (AUC), from continued non‐stationary analysis. This analysis was performed with Kubios HRV Premium Software v3.1. Using the stationary and nonstationary analysis, time domain and nonlinear domain were plotted (Time domain: SDNN: standard deviation of the NN intervals; RMSSD: root mean square of the successive differences between adjacent normal R‐R intervals; NN50: number of pairs of successive NN intervals that differ by more than 50 ms; PNN50: proportion of NN50 divided by the total number of NN intervals. Nonlinear domain: SD1: short‐term variability of NN intervals; SD2: long‐term variability of NN intervals; ApEn: Approximate entropy; SampEn: Sample entropy).

### 2.7 Cardiorespiratory coupling

The directionality and the magnitude of interactions between breathing and heart rate time series oscillations were quantified with the mutual information theory and phase models using a three‐step protocol (Zhu et al., 2013). First, we evaluated the mutual information of the phases. After, we obtained the interaction functions of the oscillators by fitting the coupled oscillator model to the data, related to phase of the cycle. As a last step, we compared the joint probability of the empirical phases with that obtained from the simulated model, in order to validate the empirical oscillator model. The data were showed as power spectral density and coupling directionality (breath to heart and heart to breath). Analysis was performed using custom Matlab routines.

### 2.8 Statistical analysis

Data are expressed as mean ± standard deviation (*SD*). All data were subjected to normality (Shapiro–Wilk) and homoscedasticity (Levene) testing. Data were evaluated using a 3 (control, EE, HIIT) × 2 (pre–post) analysis of variance (two‐way ANOVA with repeated measures), followed by Holm–Sidak post hoc analysis according to the data structure. Nonparametric variables were evaluated using Kruskal–Wallis analysis followed by Dunn's post test. A *p*‐value <.05 was considered statistically significant. All analyses were performed with GraphPad Prism 8.0 (La Jolla, CA).

## 3 RESULTS

### 3.1 Baseline cardiorespiratory parameters and changes after an acute bout of EE or HIIT exercise

At baseline, there were no significant differences between EE, HIIT, and control on cardiovascular and respiratory parameters (Table 1). Endurance exercise and HIIT significantly increased SBP, MABP, and HR (all *p* < .05; pre vs. post).

**TABLE 1:** Acute effect of endurance (EE) and high‐intensity Interval training (HIIT) on cardiovascular and ventilatory variables

	Control		EE		HIIT		Control	Endurance	HIIT
	PRE	POST	PRE	POST	PRE	POST	ΔPRE‐POST	ΔPRE‐POST	ΔPRE‐POST
SBP, mmHg	104.2 ± 10.6	97.0 ± 7.0	102.5 ± 10.4	115.6 ± 12.4*	102.5 ± 13.9	116.3 ± 13.9*	−7.3 ± 8.8	13.1 ± 9.6^+^	13.7 ± 15.1^+^
DBP, mmHg	63.2 ± 9.5	62.0 ± 4.4	68.3 ± 11.3	71.6 ± 7.6	67.5 ± 7.1	67.5 ± 7.1	−1.3 ± 7.2	3.38 ± 5.5	0.0 ± 5.4
PP, mmHg	41.0 ± 8.1	35.0 ± 7.5	34.3 ± 7.3	44.0 ± 11.1	35.0 ± 9.3	48.8 ± 11.3*	−6.0 ± 9.8	9.8 ± 6.5^+^	13.8 ± 14.1^+^
MABP, mmHg	76.2 ± 9.1	72.3 ± 4.1	78.9 ± 10.3	85.4 ± 7.8*	78.4 ± 8.8	82.9 ± 8.4*	−3.2 ± 6.2	6.6 ± 6.4^+^	4.5 ± 7.0^+^
HR, bpm	75.5 ± 13.4	71.8 ± 12.6	75.5 ± 20.3	112.3 ± 11.2*	68.3 ± 12.9	125.8 ± 13.1*	−1.63 ± 9.1	37.25 ± 18.2^+^	57.5 ± 14.3^+^ ^,^ ^†^
SpO_2_, %	97.6 ± 1.1	96.9 ± 1.1	97.6 ± 1.5	96.6 ± 1.3	97.5 ± 1.4	96.1 ± 1.5*	−0.8 ± 1.2	−1.0 ± 1.9	−1.4 ± 0.9
PEF, L/min	9.5 ± 2.2	9.5 ± 2.1	8.6 ± 2.1	8.9 ± 2.2	9.0 ± 2.1	8.9 ± 2.1	0.0 ± 0.7	0.3 ± 0.7	−0.0 ± 0.9
PIF, L/min	6.7 ± 1.8	6.7 ± 1.3	6.3 ± 1.7	6.5 ± 1.5	6.4 ± 1.5	6.6 ± 1.6	0.1 ± 0.9	0.2 ± 0.6	0.3 ± 0.9
REV, L	1.4 ± 0.3	1.3 ± 0.3	1.2 ± 0.6	1.2 ± 0.2	1.4 ± 0.2	1.4 ± 0.2	−0.2 ± 0.2	0.1 ± 0.3	−0.1 ± 0.3
VC, L	5.0 ± 0.7	4.9 ± 0.7	4.9 ± 0.8	4.9 ± 0.6	4.8 ± 0.6	4.9 ± 0.6	−0.1 ± 0.3	0.0 ± 0.2	0.1 ± 0.2

Note

Data are showed as mean ± Standard deviation (*SD*).

Abbreviations: DBP, diastolic blood pressure; HR, heart rate; MABP, mean arterial blood pressure; PEF, peak expiratory flow; PIF, peak inspiratory flow; PP, pulse pressure; REV, reserve expiratory volume; SBP, systolic blood pressure; SpO_2_, oxygen saturation; VC, vital capacity.

Two‐way ANOVA with repeated measures, following Holm–Sidak post hoc, *n* = 8.

* *p* < .05, vs. PRE condition;

^+^ *p* < .05, vs. ΔPRE–POST Control;

^†^ *p* < .05, vs. ΔPRE–POST Endurance.

### 3.2 Acute effects of EE and HIIT exercise bouts on pulmonary function

**FIGURE 2:**
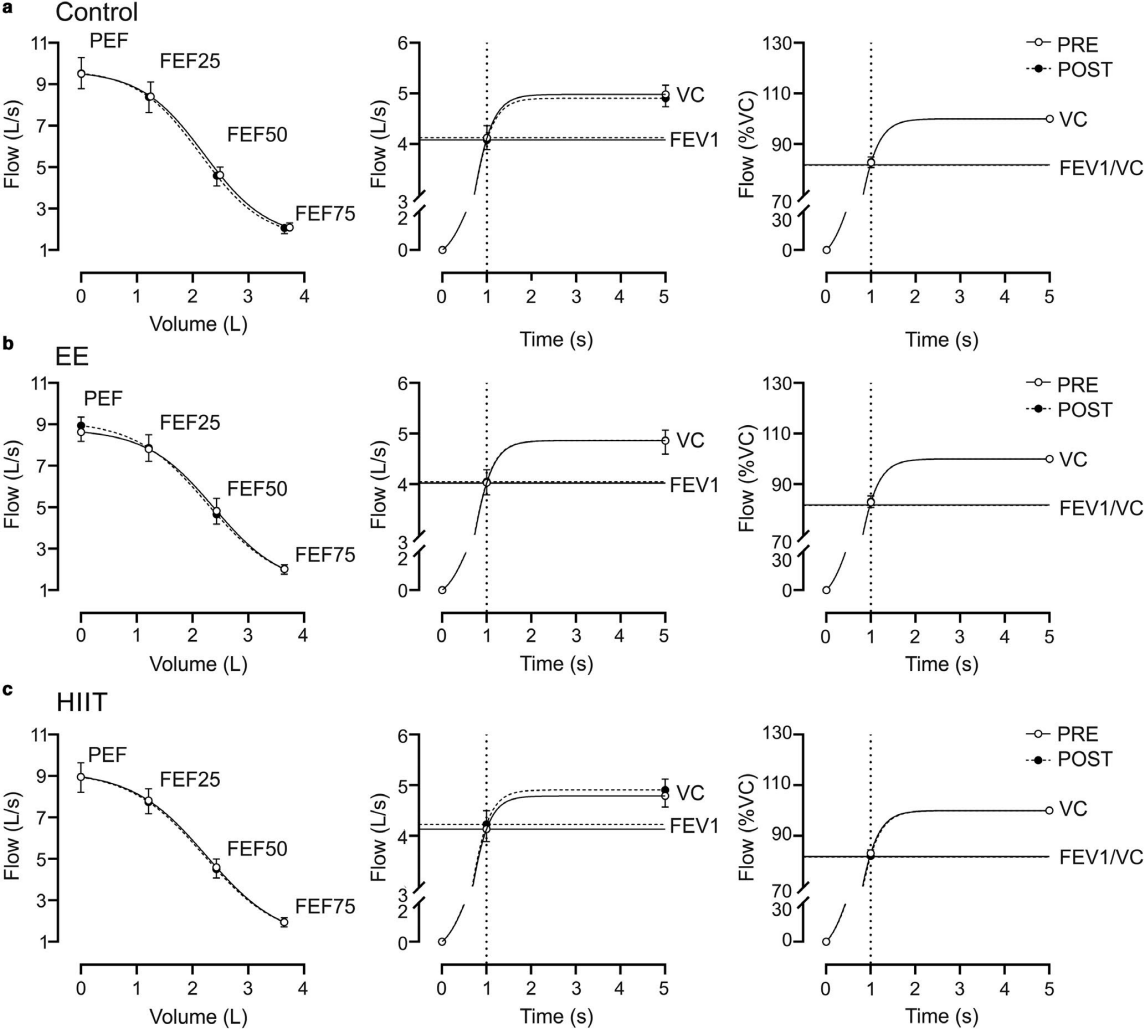
Acute effect of high‐intensity interval training and endurance exercise on pulmonary function. Summary data of pulmonary flow recording during forced expiration test. (a–c) Control, EE and HIIT, respectively, showed similar values of peak expiratory flow (PEF), forced expiratory volume at 1‐s (FEV1), forced expiratory flow at 25% (FEF 25), 50% (FEF 50) and 75% (FEF 75) of vital capacity (VC), FEF and FEV1/VC during control patients. Two‐way ANOVA with repeated measures followed by Holm‐Sidak post‐hoc test, median ± min‐max, *n* = 8

Endurance exercise and HIIT sessions did not induce significant changes on FEV1, FEV1/VC, FEF 25, FEF, or FEF 75 (*p* > .05; pre vs. post) (Figure 2). In addition, no significant differences were found on pulmonary function (pre–post) between EE, HIIT, and control conditions (Table 1 and Figure 2).

### 3.3 Acute effects of endurance and HIIT exercise bouts on heart rate variability

**FIGURE 3:**
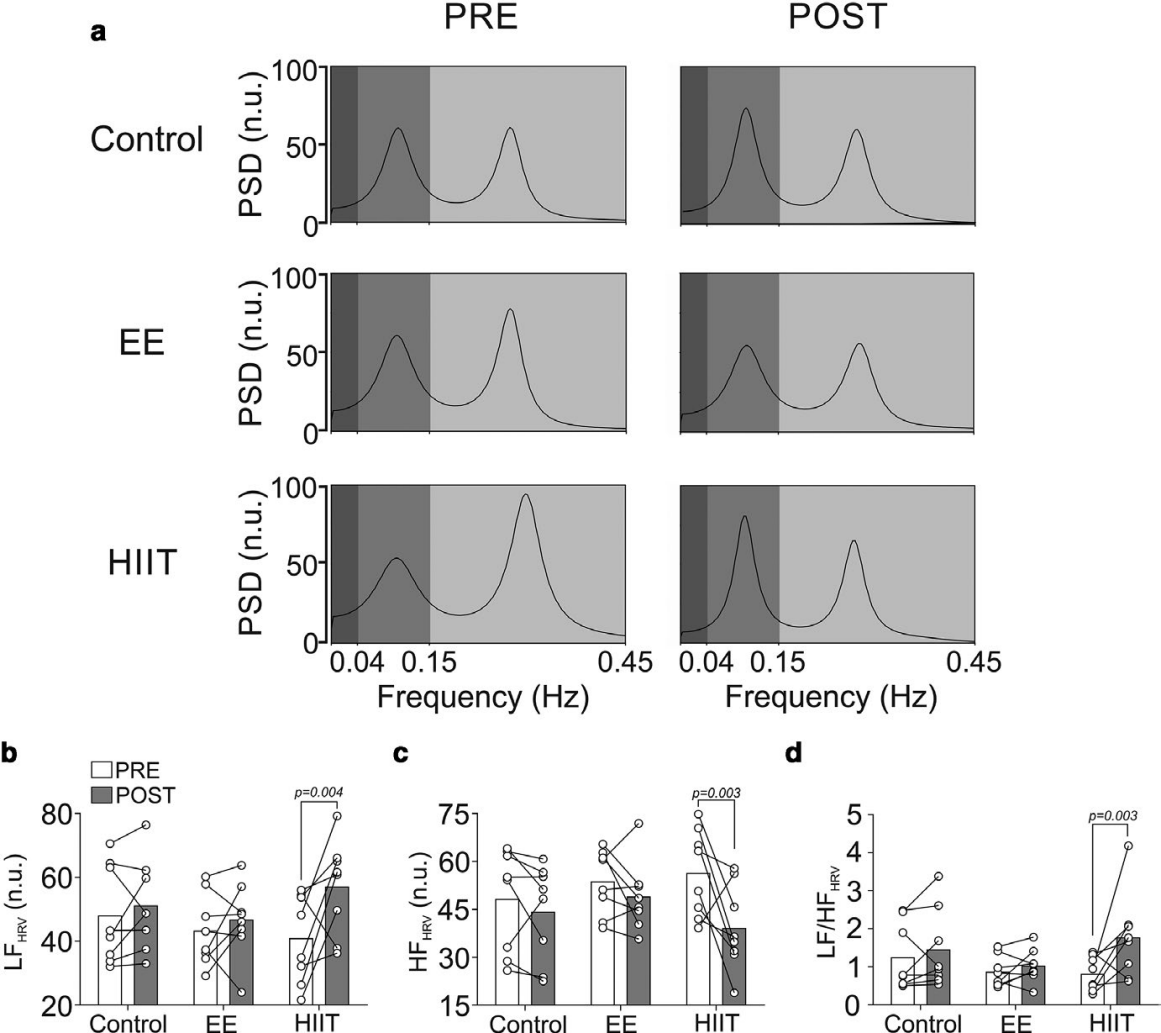
Acute effect of high‐intensity exercise training and endurance exercise on heart rate variability alterations in healthy individuals. (a) Representative spectrums of heart rate variability (HRV) in control conditions, EE and HIIT. Note that HIIT has an increase of low frequency (LF) of HRV, a decrease of high frequency (HF) of HRV and, consequently, an increase of LF/HF ratio. (b‐d) Summary quantification of LF, HF and LF/HF ratio of HRV, respectively. Two‐way ANOVA with repeated measures followed by Holm‐Sidak post‐hoc test, median ± min‐max, *n* = 8

An acute EE session failed to induce significant changes in LF_HRV_, HF_HRV_, and LF/HF_HRV_ ratio (all *p* < .05; pre vs. post) (Figure 3). However, total spectral power was significantly reduced after EE (*p* < .05; pre vs. post) (Table 2). In contrast, HIIT induces significant changes in HRV spectral data. Indeed, LF_HRV_ was increased while HF_HRV_ was reduced by HIIT (all *p* < .05; pre vs. post) (Figure 3, Table 2). Accordingly, the LF/HF_HRV_ ratio was increased after HIIT protocol (*p* < .05; pre vs. post) (Figure 3). Also, HRV total spectral power was reduced by HIIT (Table 2). Endurance exercise and HIIT showed a significant decrease in time domain variables (SDNN; RMSSD, NN50, pNN50, *p* < .05; pre vs. post) (Table 2). In addition, SD1 and SD2 were significantly decreased after EE and HIIT (Table 2).

**TABLE 2:** Acute effect of endurance (EE) and high‐intensity Interval training (HIIT) on heart rate variability (HRV) alterations

	Control		EE		HIIT		Control	Endurance	HIIT
	PRE	POST	PRE	POST	PRE	POST	ΔPRE‐POST	ΔPRE‐POST	ΔPRE‐POST
Frequency domain									
VLF, ms^2^	168.6 ± 80.0	142.3 ± 65.4	169.5 ± 131.1	125.9 ± 113.7	204.6 ± 125	50.9 * ± 44.4	26.4 ± 30.1	43.6 ± 50.8^+^	153.8 ± 100.8^+^ ^,^ ^†^
Total Power, ms^2^	1782.6 ± 1,054.6	1879.5 ± 1,108.1	2,878.9 ± 2,440.8^@^	1813.1 ± 1,720.5*	2,565 ± 1591	584.5 ± 502.7*	−96.9 ± 986.3	1,065.8 ± 873.1	1980.5 ± 1^,^190.2^+^
LF, ms^2^	896.0 ± 699.2	993.4 ± 722.6	1529.4 ± 1,411.9^@^	995.5 ± 977.9*	1,282.1 ± 781.6	370.8 ± 299.5*	−97.4 ± 320.7	533.9 ± 572.3^+^	911.4 ± 583.4^+^
HF, ms^2^	717.1 ± 376.6	743.1 ± 490.9	1,187.8 ± 949.5^@^	691.3 ± 677.1*	1,291.5 ± 653.2^@^	162.0 ± 183.9*	−26 ± 674.9	491.5 ± 325.7^+^	1^,^129.5 ± 532.7^+^ ^,^ ^†^
LF/HF	1.5 ± 1.0	1.5 ± 0.9	1.2 ± 0.6	1.6 ± 0.5	1.0 ± 0.3	3.2 ± 1.9*	0.1 ± 1.1	−0.3 ± 0.8^+^	−2.2 ± 1.8^+^ ^,^ ^†^
LF, *n*.u	54.3 ± 15.8	55.9 ± 14.0	52.4 ± 12.6	59.0 ± 9.3	48.8 ± 8.4	72.8 ± 11.2*	−1.6 ± 14.2	−6.6 ± 15.8	−24.1 ± 11.1^+^ ^,^ ^†^
HF, *n*.u	45.7 ± 15.9	44.0 ± 14.0	47.6 ± 12.6	40.9 ± 9.3	51.2 ± 8.4	27.1 ± 11.2*	1.6 ± 14.1	6.7 ± 15.9	24.1 ± 11.2^+^ ^,^ ^†^
Time domain									
Mean R‐R	884.4 ± 140.4	911.0 ± 134.5	864.5 ± 156.7	760.15 ± 96.18	877.5 ± 124.4	682.8 ± 50.8	38.8 ± 36.3	109.6 ± 68.4	194.6 ± 89.3
Mean HR	70.3 ± 10.1	67.9 ± 9.4	72.1 ± 12.6	80.7 ± 10.7	70.5 ± 10.2	88.9 ± 6.6	3.4 ± 3.1	9.4 ± 4.1	18.3 ± 5.5
SDNN	42.7 ± 10.5	43.3 ± 12.7	50.7 ± 21.2^@^	39.4 ± 18.0*	52.3 ± 13.8^@^	22.4 ± 9.7*	−0.6 ± 9.2	11.2 ± 7.9^+^	29.9 ± 9.9^+^ ^,^ ^†^
RMSSD	40.7 ± 11.2	44.2 ± 16.3	49.5 ± 19.7	34.1 ± 15.9*	51.3 ± 17.9	15.9 ± 8.6*	−3.5 ± 20.3	15.4^+^ ± 6.7^+^	35.4 ± 14^+^ ^,^ ^†^
NN50	238.1 ± 122.6	252.5 ± 156.9	324.8 ± 203.4	159.5 ± 165.6*	356.5 ± 174.8	33.1 ± 52.1*	−14.4 ± 208.8	165.3 ± 125.6	323.4 ± 137.4^+^
pNN50	18.1 ± 10.2	19.9 ± 13.4	24.8 ± 16.4	10.4 ± 11.2*	26.5 ± 13.2	1.9 ± 3.0*	−1.9 ± 17.1	14.5 ± 11.4	24.6 ± 11.3^+^
Nonlinear domain									
SD1, ms	28.8 ± 8.0	31.2 ± 11.6	35.1 ± 13.9	24.1 ± 11.3*	36.3 ± 12.6	11.2 ± 6.1*	−2.5 ± 14.4	10.9 ± 4.7^+^	25.1 ± 9.9^+^ ^,^ ^†^
SD2, ms	52.7 ± 14.3	52.2 ± 15.3	62.1 ± 27.0	50.1 ± 23.3*	61.5 ± 20.3	29.5 ± 12.5*	0.6 ± 7.8	12.3 ± 10.6	32 ± 14.6^+^ ^,^ ^†^
ApEn	1.5 ± 0.1	1.5 ± 0.1	1.4 ± 0.1	1.3 ± 0.2	1.5 ± 0.1	1.4 ± 0.1	0.1 ± 0.2	0.1 ± 0.1	0.1 ± 0.2
SampEn	1.7 ± 0.3	1.7 ± 0.2	1.7 ± 0.2	1.4 ± 0.2	1.7 ± 0.1	7.3 ± 16.3	0.1 ± 0.4	0.3 ± 0.2	−5.6 ± 16.2

Note

Data are showed as mean ± Standard deviation (*SD*).

Abbreviations: ApEn, Approximate entropy; HF, High frequency component of HRV; LF, low frequency component of HRV; NN50, number of pairs of successive NN intervals that differ by more than 50 ms; PNN50, proportion of NN50 divided by the total number of NN intervals; RMSSD, Root mean square of the successive differences between adjacent normal R‐R intervals; SampEn, Sample entropy; SD1, short‐term variability of NN intervals; SD2, long‐term variability of NN intervals; SDNN, Standard deviation of the NN intervals; VLF, very low frequency component of HRV.

* *p* < .05. Two‐way ANOVA with repeated measures, following Holm–Sidak post hoc, *n* = 8. *p* < .05, vs. PRE condition;

^+^ *p* < .05, vs. ΔPRE–POST Control;

^†^ *p* < .05, vs. ΔPRE‐POST Endurance;

^@^ *p* < .05 vs. PRE Control Condition.

### 3.4 Acute effects of EE and HIIT exercise bouts on autonomic disturbances induced by orthostatic sit‐up test

**FIGURE 4:**
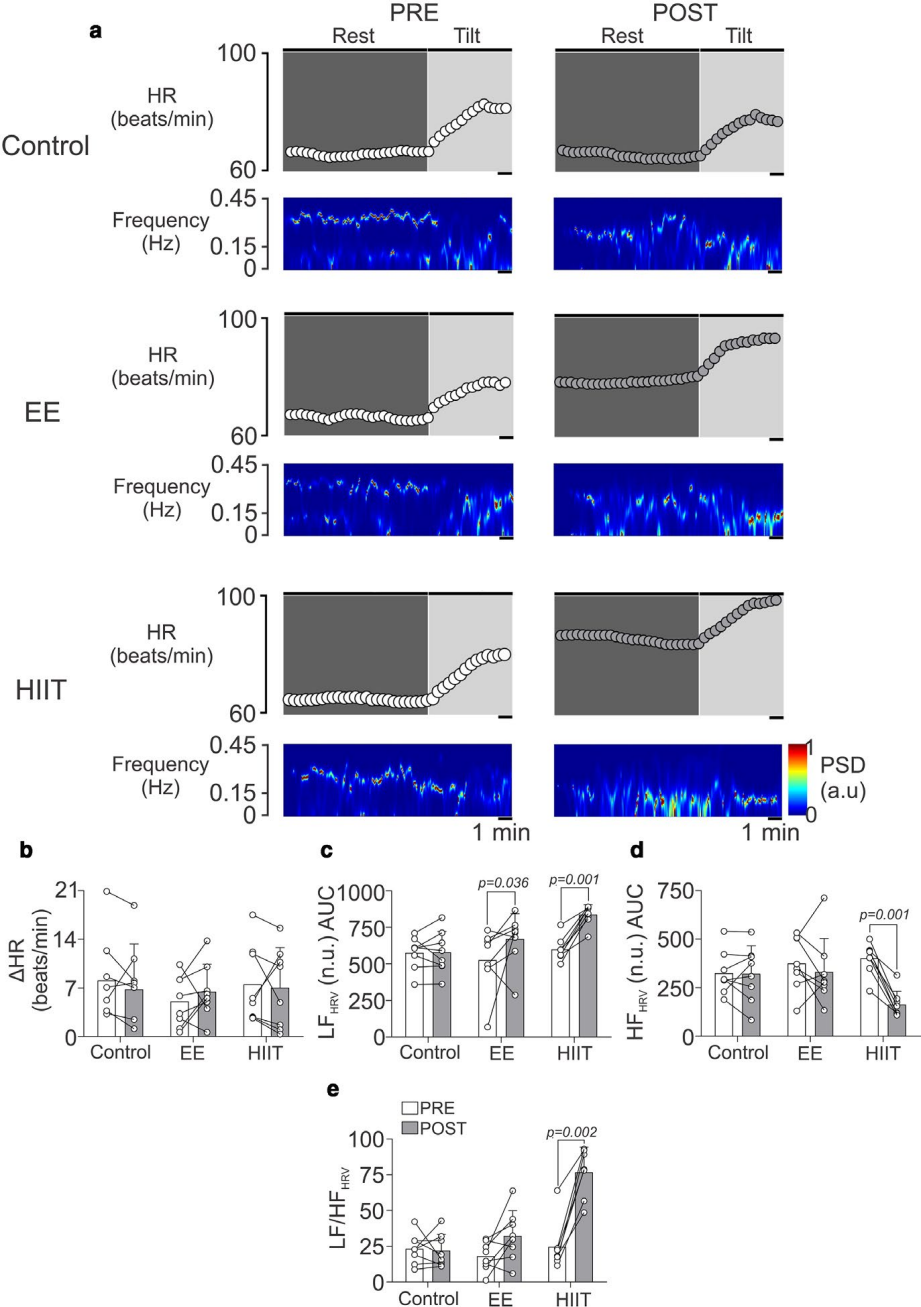
Single bout of high‐intensity exercise training elicits sympathoexcitation in healthy individuals. (a) Representative recording of heart rate (HR) and power spectral density (PSD) of non‐stationary analysis of HR with 2‐s resolution (time varying domain of HR variability) during tilt. Note that post‐HIIT the low frequency (LF) component is increased and high frequency (HF) component is decreased. Control and endurance exercise did not show significant differences (b) Summary data of △HR between before and after tilt test. (c–e) Summary data of LF, HF and LF/HF ratio of HRV non‐stationary analysis, respectively. Twoway ANOVA with repeated measures followed by Holm‐Sidak post‐hoc test. Values are median ± min‐max *n* = 8

Using nonstationary analysis of HRV during the orthostatic sit‐up test, EE and HIIT were found to produce an increase in LF_HRV_ (*p* < .05; pre vs. post). However, only HIIT elicited a significant decrease in HF_HRV_ (*p* < .05; pre vs. post) (Figure 4). The control protocol had no effect on HRV time‐varying domain variables. In addition, there were no significant differences between experimental interventions at any time regarding HR response (ΔHR) during the orthostatic sit‐up test (Figure 4). Total power, LF, and HF row data were significant different between EE and Control PRE condition. Moreover, HF row data were different between HIIT and Control PRE condition (Table 3). Regarding the time domain, we observed that at the preexercise condition, the HIIT protocol led to a significant increase in SDNN, RMSSD, NN50, and pNN50, which was not observed in the control and EE protocols (Table 3). Also, both EE and HIIT led to a significant decrease in long‐term variability and sample entropy (*p* < .05; pre vs. post) (Table 3).

**TABLE 3:** Acute effect of endurance (EE) and high‐intensity Interval training (HIIT) on heart rate variability (HRV) alterations following orthostatic sit‐up test

	Control				EE				HIIT			
	PRE		POST		PRE		POST		PRE		POST	
	Rest	SUT	Rest	SUT	Rest	SUT	Rest	SUT	Rest	SUT	Rest	SUT
Frequency domain												
VLF, ms^2^	188.9 ± 178.9	345.4 ± 265.7	155.1 ± 109.7	1997.1 ± 4,893.4	317 ± 391.7	394.6 ± 412.3	381.1 ± 719.1	592.5 ± 1,016.3	3,582.3 ± 8,371.9	701.4 ± 838.2	40.5 ± 38.3	51.9 ± 48.9
LF, ms^2^	1,207.5 ± 1,260.2	1676 ± 1,471.2	1,325.5 ± 1,640.4	3,341.5 ± 4,012.4	3,373.3 ± 4,038.2	3,006.6 ± 3,687.8	3,537.4 ± 6,326.2	4,251.4 ± 5,056.7	3,018.3 ± 2,856.8	4,063.6 ± 3,796.7	229.7 ± 198.8	875.2 ± 877.4
HF, ms^2^	1,194.2 ± 828.8	1,325 ± 1,560.9	1534 ± 2,694.2	1,390.3 ± 1583.2	2,533.4 ± 3,629	1979.1 ± 2,222.1	3,077.2 ± 6,277.2	2026.2 ± 2,794.8	4,864 ± 9,380.2	2,787 ± 3,621.3	284.3 ± 541.1	118.6 ± 176.8
LF/HF	1.4 ± 1.0	3.0 ± 1.8	1.8 ± 1.4	8.2 ± 16.2	2.3 ± 2.9	1.9 ± 1.1	2.0 ± 1.7	5.4 ± 4.3	3.6 ± 7.6	2.8 ± 2.4	2.3 ± 1.7	12 ± 10.8*
LF, *n*.u	47.6 ± 18.9	63.6 ± 12.6	51.4 ± 16.2	64.2 ± 16	51.8 ± 20.2	53.9 ± 17.4	53.1 ± 9.4	67.2 ± 18.6	43 ± 20.3	60.8 ± 9.4	56.8 ± 21.2	84.7 ± 7*
HF, *n*.u	52.4 ± 18.9	36.4 ± 12.6	48.6± 16.2	35.8 ± 16	48.2 ± 20.2	46.1 ± 17.4	46.9 ± 9.4	32.8 ± 18.6	57 ± 20.3	39.2 ± 9.4	43.2 ± 21.2	15.3 ± 7*
Time domain												
Mean R‐R	933.6 ± 157.8	802.8 ± 126.1	951.7 ± 140.1	826.4 ± 146.0	890.0 ± 174.2	803.2 ± 139.8	785.9 ± 96.0	719.9 ± 111.6	904.7 ± 126.3	814.2 ± 131.3	708.2 ± 46.8	643.8 ± 70.2
Mean HR	66.1 ± 11.1	77.0 ± 10.7	64.6 ± 9.7	75.1 ± 11.9	70.1 ± 13.4	77.6 ± 12.8	77.8 ± 9.8	86.0 ± 13.8	67.9 ± 9.2	76.3 ± 12.7	85.3 ± 5.4	94.7 ± 10.3
SDNN	42.1 ± 13.2	38.8 ± 7.8	41.8 ± 9.6	40.5 ± 10.1	49.5 ± 21.1	47.9 ± 17.1	33.3 ± 18.4	41.3 ± 15.5	55.8 ± 16.6	46.4 ± 11.5*	21.6 ± 12.6	26.8 ± 10.1
RMSSD	43.6 ± 14.5	31.7 ± 9.8	44.9 ± 14.5	36.8 ± 14.7	48.4 ± 20.6	43.7 ± 15.7	29.5 ± 16.2	36.6 ± 14.8	57 ± 21.9	38.7 ± 13.4*	17.1 ± 11.3	15.9 ± 8.7
NN50	64.3 ± 30.9	41.1 ± 29.4	63.9 ± 42	51.6 ± 35	83.3 ± 57.5	75.9 ± 40.8	35.8 ± 40.9	36.7 ± 39.4	102.2 ± 45.6	60 ± 35.6*	11.4 ± 19.9	9.3 ± 16
pNN50	20.4 ± 10.7	11.9 ± 9.6	21.2 ± 15.2	15.4 ± 11.6	26 ± 20.1	21.5 ± 12.9	9.8 ± 11.8	8.9 ± 9.6	31.8 ± 14.7	16.6 ± 9.8*	2.7 ± 4.7	2.1 ± 3.7
Nonlinear domain												
SD1, ms	30.9 ± 10.3	22.4 ± 7	31.8 ± 10.3	26 ± 10.4	34.3 ± 14.6	31 ± 11.1	20.9 ± 11.5	21.7 ± 10.4	40.4 ± 15.5	27.4 ± 9.5*	12.1 ± 8	11.2 ± 6.2
SD2, ms	50.5 ± 17.2	49.8 ± 10	49.3 ± 11.8	50.4 ± 12.9	60.8 ± 26.7	60 ± 22.2	42 ± 23.5	54.1 ± 19.8*	67.5 ± 18.6	59.4 ± 14.3	27.9 ± 16.2	36 ± 13.1
ApEn	1.1 ± 0.1	1.1 ± 0.1	1.1 ± 0.1	1.1 ± 0.1	1.1 ± 0.1	1.1 ± 0.1	1.1 ± 0.1	1.1 ± 0.1	1.1 ± 0.1	1.1 ± 0.0	1.1 ± 0.1	1.1 ± 0.1
SampEn	1.7 ± 0.2	1.6 ± 0.3	1.7 ± 0.2	1.6 ± 0.2	1.7 ± 0.2	1.5 ± 0.3	1.6 ± 0.3	1.4 ± 0.2	1.7 ± 0.2	1.5 ± 0.2	1.5 ± 0.2	1.2 ± 0.1*

Note

Data are showed as mean ± Standard deviation (*SD*).

Abbreviations: ApEn: Approximate entropy; HF: High frequency component of HRV; LF: low frequency component of HRV; NN50: number of pairs of successive NN intervals that differ by more than 50 ms; PNN50: proportion of NN50 divided by the total number of NN intervals; RMSSD: Root mean square of the successive differences between adjacent normal R‐R intervals; SampEn: Sample entropy; SD1: short‐term variability of NN intervals; SD2: long‐term variability of NN intervals; SDNN: Standard deviation of the NN intervals; SUP: sit‐up test; VLF: very low frequency component of HRV.

* *p* < .05. Two‐way ANOVA with repeated measures, following Holm–Sidak post hoc, *n* = 8. *, *p* < .05, vs. Rest condition.

### 3.5 Acute effects of EE and HIIT exercise bouts on cardiorespiratory coupling

**FIGURE 5:**
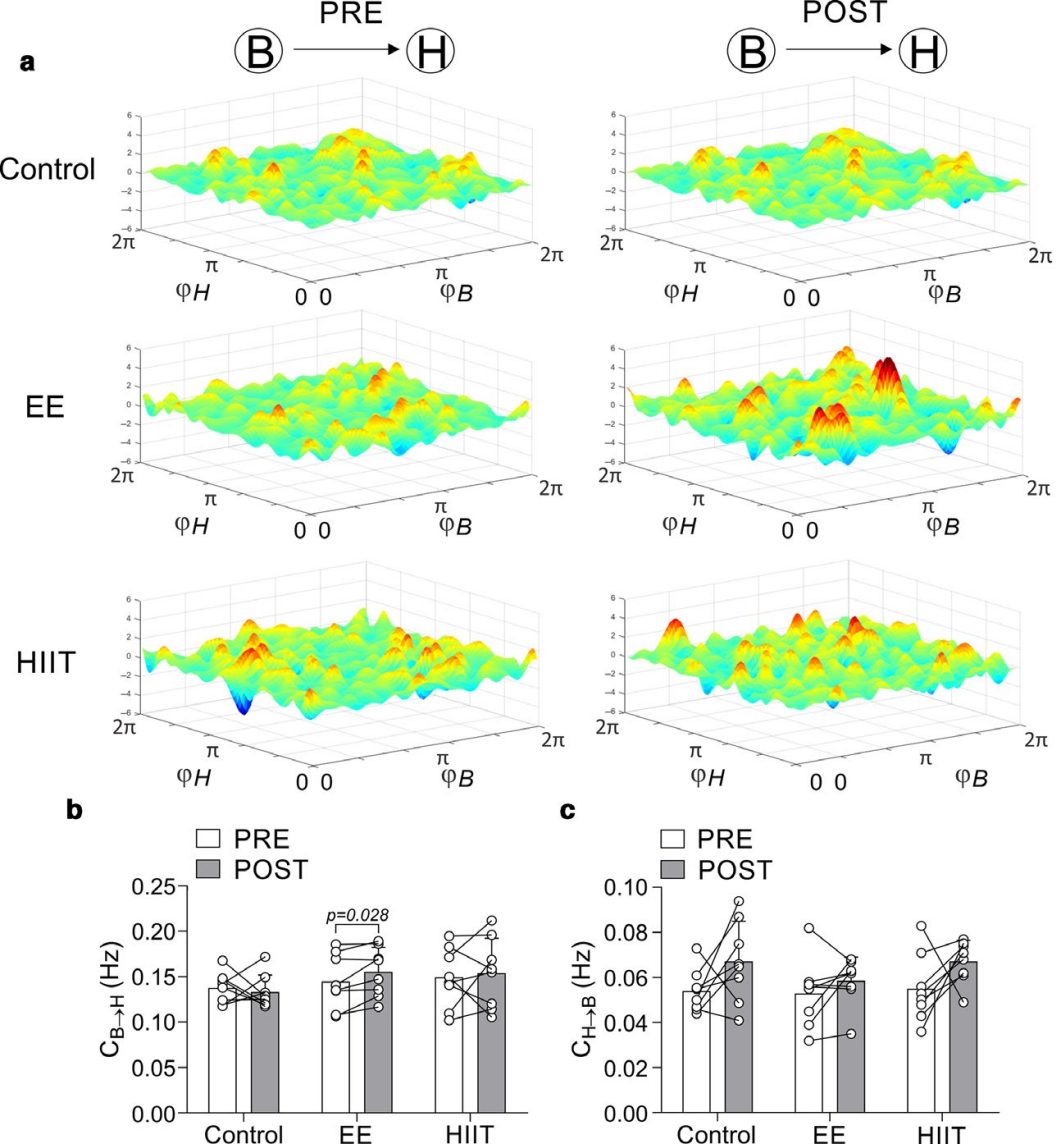
Single bout of endurance exercise increases cardiorespiratory coupling in healthy individuals. (a) Representative image of control condition, EE and HIIT before and after single bout of exercise on coupling oscillation between breath (B) and heart (H). Note that EE training results in an increase of physiological coupling after the exercise. There were no significant changes in control and HIIT. (b) Quantification of the effect of control, EE and HIIT on the coupling coefficient between B‐H (B → H). (c) Coupling coefficient between H‐B (H → B). Two‐way ANOVA with repeated measures followed by Holm‐Sidak post‐hoc test. Values are median ± min‐max, *n* = 8

Directionality analysis showed that coupling between breathing and heart rate (B → H) was superior than the one going from the heart rate to the breathing (H → B) in control conditions (Figure 5). B → H coupling but not H → B was significantly increased after EE (0.14 ± 0.03 vs. 0.16 ± 0.03 Hz, pre vs. post, respectively, *p* < .05). No effect of HIIT on B → H nor in H → B coupling was found.

## 4 DISCUSSION

The aim of the present study was to compare the acute effects of EE and HIIT on pulmonary function, autonomic balance, and CRC in healthy subjects. The main findings were: (a) neither EE nor HIIT change pulmonary function; (b) HIIT and not EE produced acute HRV changes evidenced by a decreased HF_HRV_ and an increased LF_HRV_ postexercise; (c) HIIT but not EE have detrimental effect on the normal autonomic cardiovascular adjustment to an tilt challenge; and (d) EE but not HIIT induced an acute increase on CRC. Overall, our findings suggest that, acute cardiorespiratory responses to EE and HIIT may differ between these two exercise modalities, which could have some implications to chronic exercise adaptations.

### 4.1 Effect of single bout of EE and HIIT on pulmonary function

Pulmonary function is defined as the capacity of the lungs to transfer oxygen from the air to the organism. The acute effects of exercise training on pulmonary function are controversial. While it has been reported that low‐ and high‐intensity exercise training (40% and 75% of peak work, respectively) does not compromise pulmonary function (Beck, Hyatt, Mpougas, & Scanlon, 1999; Snyder et al., 2006), it has also been observed that high‐intensity exercise could produce bronchoconstriction in subjects without history of asthma, which could negatively impact pulmonary function (Anderson, 2006). However, none of the participants showed ventilatory distress during experimental procedures. O'Kroy, Loy, and Coast (1992) proposed that pulmonary function changes after exercise could be associated with respiratory muscle fatigue, which negatively impacts vital capacity (O'Kroy et al., 1992). However, despite evidence showing the effects of low‐ and high‐intensity exercise, there are no data comparing the effects of a single bout of HIIT or EE on pulmonary function in healthy subjects. Our present data show that an acute bout of HIIT and EE (middle intensity) does not significantly change pulmonary function measured by clinical spirometry. It is worth noting that one of the more important mechanisms associated with bronchoconstriction and related‐pulmonary malfunction after acute exercise is the sympathetic/parasympathetic balance (Tipton, Kadinopoulos, de Sa, & Barwood, 2017). Although we found no changes in pulmonary function, autonomic control (i.e., HRV disturbances) was altered after HIIT but not after EE. Therefore, it is possible that alterations in autonomic control induced by HIIT may trigger pulmonary dysfunction. Thus, further studies are needed to fully elucidate the possible role of autonomic balance and HIIT on pulmonary function.

### 4.2 Effect of EE and HIIT on HRV disturbances

The autonomic nervous system plays an important role in maintaining homeostasis under normal and pathological conditions. HRV analysis has been used as an indirect surrogate of cardiac autonomic balance (Camm et al., 1996), which reflects beat‐to‐beat changes of HR, representing the sympathetic/parasympathetic interaction (Camm et al., 1996). Accordingly, HRV analysis has been used to estimate autonomic control impairment in physiological and pathophysiological conditions (Alansare et al., 2018; Andrade et al., 2017; Arce‐Alvarezet al., 2019; Henríquez, Báez, Von Oetinger, Cañas, & Ramírez, 2013; Nakamura et al., 2015; Ramírez‐Vélez et al., 2017). It has been shown that exercise training could elicit disturbances in HRV (Heffernan, Kelly, Collier, & Fernhall, 2006; Niemelä et al., 2008). Our data show that EE did not elicit HRV alterations. On striking contrast, we found that a single bout of HIIT negatively altered autonomic regulation, characterized by an increase in sympathoexcitation and parasympathetic withdrawal, determined by changes in LF and HF, respectively. This difference observed in EE compared with previous evidence (Panissa, Cal Abad, Julio, Andreato, & Franchini, 2016) could be related to the time recovery period, and to the analysis of the triggered signal (i.e., R‐R signal vs. ECG vs. SBP) (Niemelä et al., 2008). In addition, it has been documented that a single bout of HIIT could deteriorate HRV variables (Panissa et al., 2016). It is interesting to note that HRV alterations post‐HIIT could depend on training status, intensity, and duration of exercise (Seiler, Haugen, & Kuffel, 2007). Thus, our data are in accord with those of others (Panissa et al., 2016). Moreover, we observed that after the HIIT session LF_HRV_, HF_HRV_ and, consequently, the LF/HF ratio were increased, decreased, and increased, respectively.

The principal evidence regarding autonomic function alterations following a single bout of physical exercise has been established through stationary analysis (i.e., fast Fourier transform, autoregressive, among others), nevertheless here we used nonstationary analysis (time‐varying domain) to observe faster events during our experiments (Tarvainen, Georgiadis, Ranta‐Aho, & Karjalainen, 2006). To determine the effect of autonomic challenge, we induced a deregulation of sympathetic/parasympathetic balance through the orthostatic sit‐up test (Tarvainen et al., 2006). It has been shown that a single bout of EE elicited a late effect on HRV disturbances following the orthostatic sit‐up test; however, this result was obtained through stationary analysis (Martinelli et al., 2005). Our data show that an EE protocol promotes an increase in the LF_HRV_ component, without significant effects on HF_HRV_ and the LF to HF ratio during the orthostatic sit‐up test, suggesting that our endurance protocol is not sufficient to elicit a worsening alteration of autonomic deregulation induced by this test. In addition, regarding the HIIT session, our results show that high‐intensity training worsens autonomic control induced by the orthostatic sit‐up test. Indeed, we observed an increase in LF_HRV_, a decrease in HF_HRV_, and an increase in the LF/ HF ratio, after a single bout of HIIT. It is important to emphasize that ΔHR was similar between all experimental conditions, which suggests that our orthostatic sit‐up test triggered similar cardiovascular effects, but with different autonomic responses. In addition, while both stationary and nonstationary analysis show that HIIT induces autonomic deregulation, this stimulus might be insufficient to induce a deterioration of pulmonary function, considering that one of the principal mechanisms related to pulmonary function impairment following exercise training is the sympathetic/parasympathetic balance.

### 4.3 Endurances exercise promotes cardiorespiratory coupling in healthy individuals

Cardiorespiratory coupling is the interaction between respiratory drive (oscillator 1) and heart rhythm (oscillator 2), which are two nonlinear dynamic variables. It has been proposed that CRC is dependent on brainstem activity, heart time‐series, and lung capacity (Scholkmann & Wolf, 2019). It has been shown that following exercise training in normal mice, the brainstem activity is increased compared with sedentary conditions (Barna, Takakura, & Moreira, 2014). In addition, regarding evidence in humans, during moderate exercise training there is an increase in CRC determined through cross‐correlation analysis (Santos et al., 2010). However, no evidence was available showing the effects of HIIT on CRC. Our data suggest that EE could affect CRC in healthy individuals. Surprisingly, a single bout of HIIT did not affect CRC, estimated through mutual information and phase analysis, which was what we did not expect, since HIIT elicited autonomic control impairment and as previously mentioned, CRC could have influence related to autonomic regulation. Conversely, a single bout of EE elicited an increase in CRC, suggesting that endurance physical effort could have beneficial effects on coordination of these two oscillators, possibly improving energy efficiency of these two systems. In addition, CRC is strongly associated with parasympathetic modulation of the heart (Yuda et al., 2018). It is thus possible that EE could improve the parasympathetic‐lung interaction, promoting efficiency of the cardiorespiratory system in healthy individuals. However, our data not showed robust effects on parasympathetic modulation of the heart, determined by HRV alterations, after EE protocol. Then, it is possible that our technic to determine vagal control does not have a sufficient resolution level to determine little changes into parasympathetic modulation of the heart, which could be associated to changes in CRC post EE.

## 5 LIMITATIONS

Our study comprised a relatively small sample size. In addition, despite a similar number of male and female between groups, the small sample size precludes robust sex‐related conclusions. Moreover, the cross‐sectional nature of our study limits us to infer potential long‐term effects of HIIT and/or endurance exercise on pulmonary function, cardiac autonomic control, and CRC. Future investigations should address this important aspect. In addition, instead of a dysfunction capacity test using carbon monoxide, a clinical spirometry test was used in our experiments of pulmonary function, precluding attainment of clinically relevant data. On the other hand, our autonomic balance analysis was performed by HRV alterations in short‐term recordings. However, previous studies have been validated the use of short‐term records (5–10 min) for nonlinear and frequency domain of HRV analysis (Camm et al., 1996). However, and without prejudice to the foregoing, we use nonstationary analysis to discard the influence of time recording (time‐varying domain). Finally, at PRE condition some differences were observed between groups, although only in row HRV data. Therefore, considering that after normalizing the units of power spectral densities these differences were not observed, it is possible that periodic breathing and/or movement devices (Camm et al., 1996) could influence row data. Future studies may elucidate the contribution of sympathetic/parasympathetic balance and CRC elicited by different training strategies, in order to improve therapies aimed at populations with different physiological and pathophysiological conditions.

## 6 CONCLUSIONS

The present study shows that EE elicited an increase of CRC in healthy individuals, independent to parasympathetic modulation of the heart; however, an acute bout of HIIT or EE did not modify pulmonary function, as determined by clinical spirometry. In addition, a HIIT session elicited a deterioration of autonomic regulation in supine position and after the orthostatic sit‐up test, as evidenced by an increase in LF_HRV_, decrease in HF_HRV_, and increase in the LF/HF_HRV_ ratio, which suggest an overall autonomic deregulation. These findings demonstrate an acute beneficial effect of EE over HIIT in healthy individuals. While our results suggest a potential beneficial effect after only one EE session, whether this would elicit beneficial effects if repeated regularly remains unclear. If our results are confirmed by a longer, chronic study, these findings could inform those responsible for designing and implementing physical activity interventions in healthy and possibly pathophysiological conditions. Thus, future research should aim to determine the chronic effects of EE on CRC, which might establish the optimum intensity and duration of exercise for pulmonary function, autonomic regulation and CRC improvements in healthy individuals.

## CONFLICT OF INTEREST

None of the authors declare competing financial interests.

## AUTHORS' CONTRIBUTIONS

DCA and AAA performed data collection and analysis, performed interpretation of the data, and contributed to the preparation of the manuscript. DCA, AAA, FP, SU, PG, AD, CO, FP, CL, and GC performed data collection and analysis, and contributed to the preparation of the manuscript. RRC, MVM, RDR, and MI performed interpretation of the data and contributed to the preparation of the manuscript. DCA contributed to the concept of the project. DCA performed interpretation of the data and contributed to the preparation of the manuscript. All data analysis and interpretation were undertaken in the laboratory of DCA. All authors approved the final version of the manuscript.

## HUMAN ETHICS

The Universidad Mayor Ethic Committee approved the study. (Approval number #169_2019).
